# Degree sums and dense spanning trees

**DOI:** 10.1371/journal.pone.0184912

**Published:** 2017-09-19

**Authors:** Tao Li, Yingqi Gao, Qiankun Dong, Hua Wang

**Affiliations:** 1 College of Computer and Control Engineering, Nankai University, Tianjin 300071, P.R. China; 2 College of Software, Nankai University, Tianjin 300071, P.R. China; 3 Mathematical Sciences, Georgia Southern University, Statesboro, GA, 30460, United States of America; school of science, Beijing University of Chemical Technology, CHINA

## Abstract

Finding dense spanning trees (DST) in unweighted graphs is a variation of the well studied minimum spanning tree problem (MST). We utilize established mathematical properties of extremal structures with the minimum sum of distances between vertices to formulate some general conditions on the sum of vertex degrees. We analyze the performance of various combinations of these degree sum conditions in finding dense spanning subtrees and apply our approach to practical examples. After briefly describing our algorithm we also show how it can be used on variations of DST, motivated by variations of MST. Our work provide some insights on the role of various degree sums in forming dense spanning trees and hopefully lay the foundation for finding fast algorithms or heuristics for related problems.

## Introduction

### Background information

A *spanning tree* *T* of a graph *G* is a connected acyclic subgraph that contain all vertices of *G*. In the case that *G* is weighted, the classic problem of finding the *minimum spanning tree* (MST) seeks the spanning tree with the minimum weight (sum of edge weights on the spanning tree). Because of its extensive applications such as network design and cluster analysis, numerous studies have been published on the algorithms (see, for instance, [1] and the references therein) and related topics including a number of variations of MST such as the *k*-MST (finding the minimum subtree containing exactly *k* vertices), the Steiner tree problem [2], degree constrained minimum spanning tree problem [3], capacitated minimum spanning tree problem [4], MST with conflict pairs [5].

A variation of the tree with minimum weight (in weighted graphs) is the tree with minimum sum of pairwise distances between vertices (in unweighted graphs). This “sum of distances” has been a simple but interesting mathematical concept since early 20th century, but has started receiving tremendous attention in the last couple of decades as the so-called *Wiener index* [6, 7] for its applications in biochemistry:

$$\begin{array}{c} W\left(T\right)=\sum_{u,v\in V(T)}d\left(u,v\right). \end{array}$$

Here *d*(*u*, *v*) is the distance between *u* and *v*. Thus a natural variation of the MST is to find the spanning tree with the minimum Wiener index.

Extremal trees and graphs that minimize the Wiener index in various classes of graphs have been extensively studied, see [8] for an earlier informative survey and part of [9] for some recent results. One interesting observation was that the extremal structures that minimize the Wiener index usually maximize the number of subtrees (see for instance [10]). This correlation was further analyzed in [11]. The number of subtrees relates to the complexity of phylogeny reconstruction algorithms [12] and “density” of graphs [13, 14].

Intuitively, indeed a “dense” structure with many subtrees tends to minimize the sum of distances. Consequently the MST becomes finding densest (with minimum Wiener index) spanning trees (DST) in unweighted graphs. We explore the known mathematical properties of dense trees that lead to useful methods for solving DST.

### Degree sequence and the greedy tree

In the study of dense trees, trees with a given *degree sequence* (non-increasing sequence of vertex degrees) are often considered. It has been established that the *greedy tree* (Definition 1) minimizes the Wiener index and maximizes the number of subtrees among all trees with a given degree sequence. Here we use *deg*(*v*) to denote the degree of a vertex *v*.

**Definition 1 (Greedy Tree)**. *With a given degree sequence, the greedy tree is achieved through the following “greedy algorithm”:*

i Label the vertex with the largest degree as v (the root);
ii *Label the neighbors of v as v*_1_, *v*_2_, *…*, *assign the largest degrees available to them such that deg(v*_11_ *)≥deg(v*_12_ *)≥…;*
iii *Label the neighbors of* *v*_1_ *(except v) as v*_11_, *v*_12_, *…,such that they take all the largest degrees available and that deg(v*_11_ *)≥deg(* *v*_12_ *)≥…*, *then do the same for* *v*_2_, *v*_3_, *…;*
iv Repeat (iii) for all the newly labeled vertices. Always start with the neighbors of the labeled vertex with largest degree whose neighbors are not labeled yet.

Furthermore, greedy trees with different degree sequences can be compared according to their Wiener indices or numbers of subtrees. Without going into details, it is easy to see that the degree sequences (6, 5, 4, 3, 2, 2, 1, …, 1) and (5, 4, 4, 3, 3, 3, 1, …, 1) correspond to trees with same number of vertices; and it is easy to verify that the greedy tree with the first degree sequence is “denser”. Based on the simple idea of putting larger degrees closer and obtaining “better” degree sequences, in [15] an edge-swapping heuristic was presented for the DST.

In order to further explore the potential of using degrees as a credential for measuring the denseness of a spanning tree, we explore a number of conditions on the sum of vertex degrees.

### Methodology

In a recent study [16], as an effort to find dense spanning trees sum of vertex degrees is used as a possible condition. It is pointed out that finding a spanning tree *T* of a given graph *G* that maximize

$$\begin{array}{c} \sum_{uv\in E(T)}\left(deg\left(u\right)+deg\left(v\right)\right) \end{array}$$ (1)

can be handled through simple integer linear programming. Note that (1) equivalent to

$$\begin{array}{c} \sum_{v\in V(T)}{\left(deg\left(v\right)\right)}^{2}. \end{array}$$ (2)

Hence condition (1) is merely choosing degree sequences. For the same degree sequence, the condition

$$\begin{array}{c} \sum_{uv\in E(T)}\left(deg\left(u\right)+deg\left(v\right)\right)+\sum_{d(u,v)=2}\left(deg\left(u\right)+deg\left(v\right)\right) \end{array}$$ (3)

can also be easily realized through integer linear programming. This second condition takes into consideration the sum of degrees at distance 2 apart, and hence further select from spanning trees with the same degree sequence. Due to the limitations of integer linear programming, further variations of such conditions cannot be tested.

In this note, for a vector (of real numbers) $\vec{j}=\left(j_{1},j_{2},\ldots ,j_{i}\right)$, we let $C_{\vec{j}}$ be the condition

$$\begin{array}{c} C_{\vec{j}}=C\left(j_{1},j_{2},\ldots ,j_{i}\right)=C_{1,j_{1}}+C_{2,j_{2}}+\ldots +C_{i,j_{i}} \end{array}$$

where the condition *C*_*i*,*j*_ is a generalization of (1), with

$$\begin{array}{c} C_{i,j}=\sum_{d(u,v)=i}({\left(deg\left(u\right)\right)}^{j}+{\left(deg\left(v\right)\right)}^{j}). \end{array}$$

It is obvious that *C*_1,1_ is exactly (1) and *C*(1, 1) is exactly (3). We seek solutions to DST through maximizing $C_{\vec{j}}$. As an intuitive explanation, we note that maximizing such expressions finds “superior” degree sequences as discussed earlier. And among spanning trees with the same degree sequence these conditions put vertices with larger degrees closer to each other.

### Results and discussion

We will first explore performances of the proposed methodology with various choices of $\vec{j}$. First we provide a comprehensive examination, followed by some concluding remarks on possible optimal vectors $\vec{j}$. We then apply our optimized parameters in some practical examples. We also briefly describe our algorithm and mention the application of our method to variations of DST, motivated from variations of MST in the literatures. In the end we summarize our results and propose some future work.

## Performance analysis

In this section we apply various degree sum conditions and evaluate the Wiener index of the resulted spanning tree.

### Sum of degrees at distance *i*

**Fig 1:**
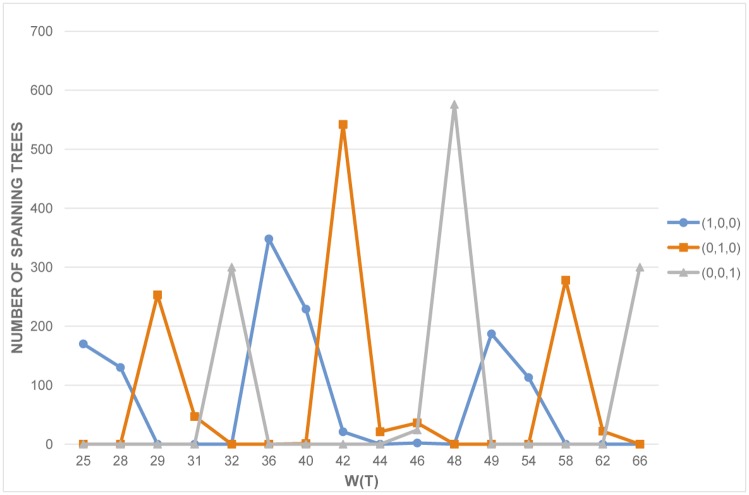
Comparison between $\vec{j}=\left(1,0,0\right),\left(0,1,0\right),\left(0,0,1\right)$.

First consider the case when $\vec{j}$ has only one nonzero entry. In what follows we let $\vec{j}=\left(1,0,0\right),\left(0,1,0\right),\left(0,0,1\right)$ and apply $C_{\vec{j}}$ to 1200 random graphs with 6, 7, or 8 vertices. The Wiener index of the selected spanning trees is evaluated and the distribution is plotted in Fig 1.

It is obvious that $\vec{j}=\left(1,0,0\right)$ performs much better than the other two. We conclude that at least for small graphs, the adjacent degree sum condition (corresponding to ($\vec{j}=\left(1,0,0\right)$) outperforms any other single sum of distances.

**Fig 2:**
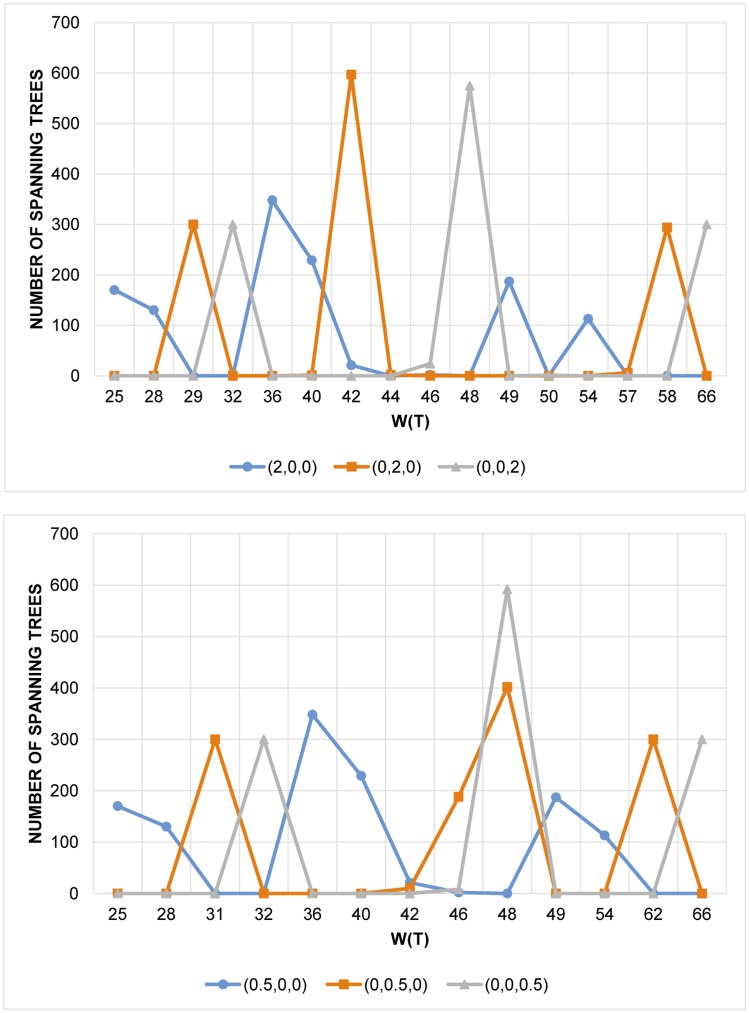
Comparison between $\vec{j}=\left(2,0,0\right),\left(0,2,0\right),\left(0,0,2\right)$ (top) and $\vec{j}=\left(0.5,0,0\right),\left(0,0.5,0\right),\left(0,0,0.5\right)$ (bottom).

Sometimes the exponent also makes a difference, for instance, for the same set of random graphs the conditions with $\vec{j}=\left(2,0,0\right),\left(0,2,0\right),\left(0,0,2\right)$ generally performs better than the ones with $\vec{j}=\left(0.5,0,0\right),\left(0,0.5,0\right),\left(0,0,0.5\right)$, as shown in Fig 2. On the other hand, exponents higher than 2 do not seem to make a difference in the result.

### Sum of degrees at different distances

**Fig 3:**
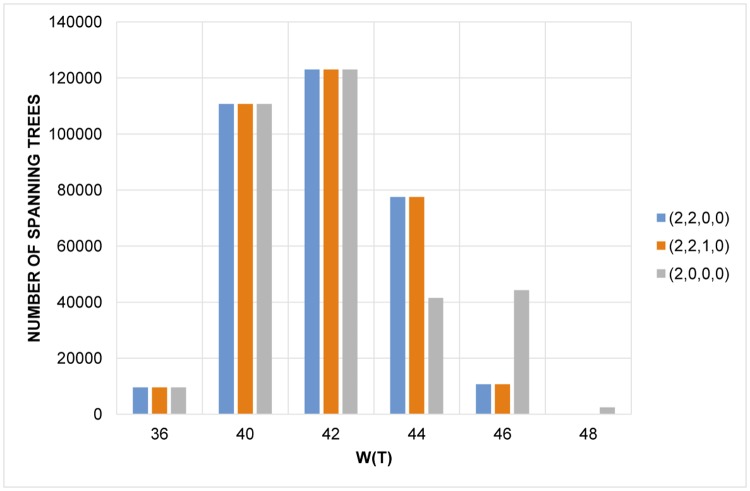
Comparison between $\vec{j}=\left(2,2,0,0),\;(2,2,1,0),\;\text{and}\;(2,0,0,0\right)$.

In the case of a sparse graph, it is likely that the adjacent degree sum condition is no longer sufficient to find the best solution. For instance, the conditions with $\vec{j}=\left(2,2,0,0)\;\text{and}\;(2,2,1,0\right)$ outperform that with $\vec{j}=\left(2,0,0,0\right)$ when applied to the set of all (labeled) random graphs on 7 vertices and 10 edges, as plotted in Fig 3.

**Fig 4:**
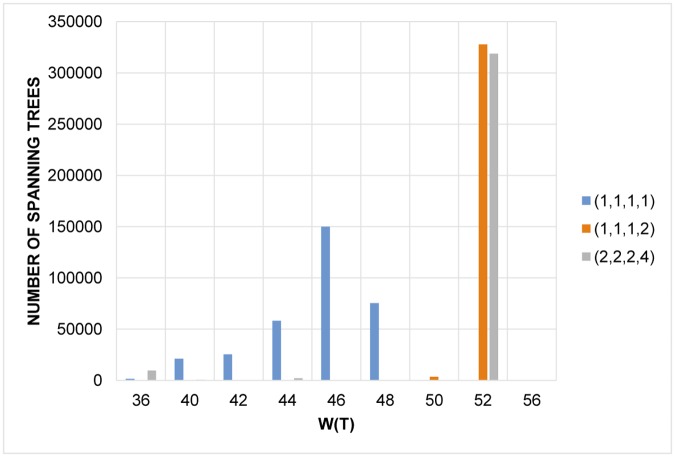
Statistics corresponding to conditions with $\vec{j}=\left(1,1,1,1),\;(1,1,1,2),\;\text{and}\;(2,2,2,4\right)$.

At least for small graphs it does not seem to be beneficial to include sum of nonzero power of vertex degrees at distance 4 or more, as shown in Fig 4 where the conditions with $\vec{j}=\left(1,1,1,1),\;(1,1,1,2),\;(2,2,2,4\right)$ do not provide any better result than those shown in Fig 3.

### Optimal criteria

**Fig 5:**
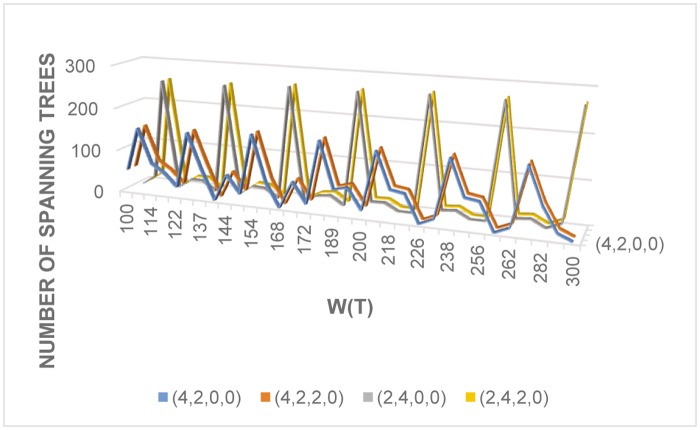
Statistics corresponding to conditions with $\vec{j}=\left(4,2,0,0),\;(4,2,2,0),\;(2,4,0,0),\;\text{and}\;(2,4,2,0\right)$.

Based on these experiments, selecting from over 30 variations of $\vec{j}$, we have narrowed our possible optimal conditions to a few vectors. We apply these particular $\vec{j}$ to a set of over 2000 large random graphs on 11, 12, 13, 14, 15, 16, 17 vertices, the performance is shown below. As one can see (from Fig 5) the conditions corresponding to $\vec{j}=\left(4,2,0,0),\;(4,2,2,0\right)$ outperform conditions corresponding to $\vec{j}=\left(2,4,0,0),\;(2,4,2,0\right)$, etc.

### Discussion

We have systematically analyzed the performance of various degree sum conditions to find dense spanning trees. When only one degree sum has nonzero power, the adjacent degree sum condition greatly outperforms the others (Fig 1), as one would expect. Furthermore, using larger exponents generally result in better performance (Fig 2).

On the other hand, since the adjacent degree sum condition is equivalent to simply the sum of squares of degrees, it is obvious that including multiple degree sums in the condition should lead to better result. This fact is verified in (Fig 3). However, when a star (generally considered as the densest tree) or “the second densest” structure exists as a spanning subgraph, the adjacent degree sum condition does always find the densest spanning tree. As in the case of graphs with 7 (labeled) vertices, all 9555 cases of the spanning star and 110691 cases of a spanning *T*_1_ (a tree with degree sequence (5, 2, 1, 1, 1, 1, 1)) are found through the adjacent degree sum condition.

This is because of the uniqueness of these dense spanning trees (and hence can be identified with the adjacent degree sum condition alone) given their degree sequences. This is formally stated below.

**Proposition 1.** *For a graph on* *n* *vertices*, *if the densest spanning tree is a star (with degree sequence* (*n* − 1, 1, …, 1)*) or a tree with degree sequence* (*n* − 2, 2, 1, …, 1), *using the adjacent degree sum condition will always find these spanning trees.*

While in theory we believe that conditions involving five or more degree sums could be useful in very large graphs, it seems that (from our collected data) in practice (when all graphs are of “reasonable size”) the conditions $C_{\vec{j}}$ with $\vec{j}=\left(4,2,0,0),\;(4,2,2,0)\;\text{or}\;(4,2,2,2\right)$ result in the densest spanning trees.

## Exemplary applications

We now apply our established “optimal conditions” with $\vec{j}=\left(4,2,0,0),\;(4,2,2,0)\;\text{and}\;(4,2,2,2\right)$ to find dense spanning trees in some practical applications.

**Fig 6:**
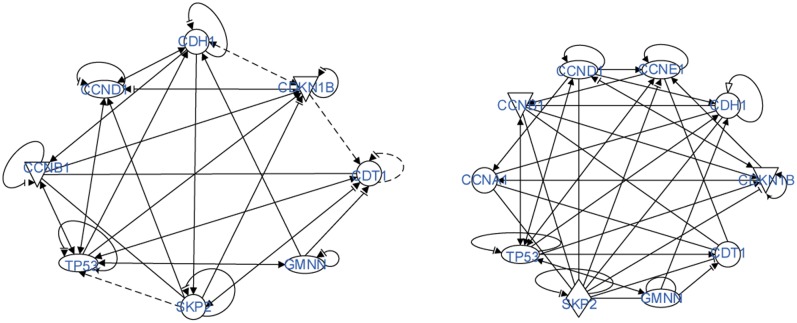
The 8-gene and 10-gene models of molecular circuits.

First, in Fig 6 we have two models of molecular circuits of cell cycle control, established by using QIAGEN’s Ingenuity Pathway Analysis (IPA, QIAGEN Redwood City, www.qiagen.com/ingenuity). The originally generated network contain many more proteins including key proteins in regulating cell cycle and with extremely high relevance in human cancers, IPA analyses was used to represent the protein-interaction network by fewer proteins, which serve as molecular hubs for the circuits of cell cycle control.

**Fig 7:**
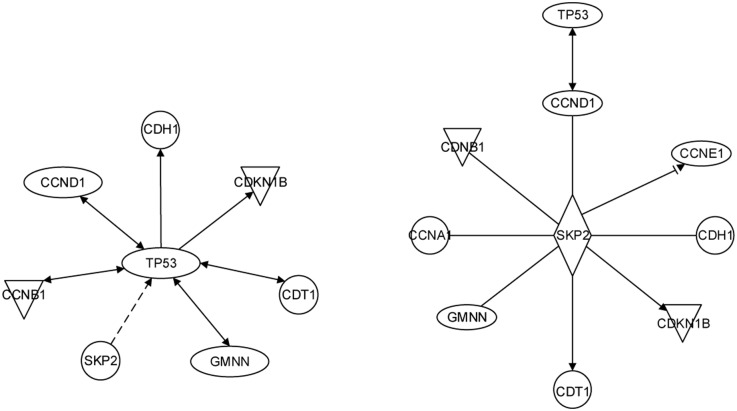
Dense spanning trees in the 8-gene and 10-gene models.

Conditions with $\vec{j}=\left(4,2,0,0)\;\text{or}\;(4,2,2,0)\;\text{or}\;(4,2,2,2\right)$ lead to the same results shown in Fig 7.

As one can see from the result, our dense spanning trees identifies the key proteins, evidently TP53 in the 8-gene model and SKP2 in the 10-gene model. This finding is consistent with the biological findings that confirms the importance of these two genes in cell cycle control.

**Fig 8:**
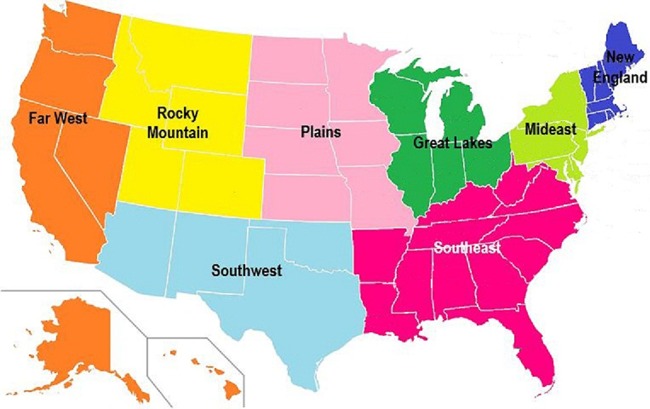
A map of the United States with 8 regions.

**Fig 9:**
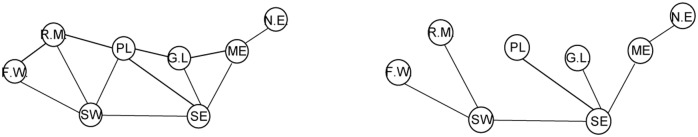
The graph representation (on the left) and the dense spanning tree (on the right).

Next, Fig 8 shows the eight regions of mainland United States, to its graph representation we apply our “optimal conditions” and find the same densest spanning tree that “centers” at the Southeast (Fig 9).

The “center position” of the Southeast region on this map and the corresponding dense spanning tree is rather obvious from the fact that it is adjacent or close to the most number of regions. This trivial observation, however, does lend support to many observations where the Southeast stands out from the rest of the country. For instance, Table 1 shows the number of incidences of an infectious disease Hepatitis B in each region in the years 2010 through 2014, published by the U.S. Department of Health and Human Services, Center for Disease Control and Prevention [17]. It is easy to see that the Southeast region has the largest infected population.

**Table 1:** Number of incidences of Hepatitis B of each region in the US.

	2010	2011	2012	2013	2014
SOUTHEAST	1272	1276	1342	1498	1389
MIDEAST	355	366	309	282	288
NEW ENGLAND	55	97	105	94	59
GREAT LAKES	481	353	457	482	416
PLAINS	130	124	55	116	78
SOUTHWEST	540	328	266	213	212
ROCKY MOUNTAINS	63	36	44	46	46
FAR WEST	385	253	223	208	174

## Algorithm and similar problems

In this section we briefly describe our algorithm (Algorithm 1). Note that our goal is to analyze the effectiveness of each degree sum condition and we make no attempt in optimizing the algorithm.

**Algorithm 1:** Finding the densest spanning tree and its Wiener index.

 **input:** A connected graph *G* on *n* vertices; positive integer *i*, vector $\vec{j}$

 **output:** The densest spanning tree (under condition $C_{\vec{j}}$) and its Wiener index

**1** initialization;

**2 foreach** *subgraph* *S* *of* *G* *with* *n* − 1 *edges* **do** save *S* to the set S if *S* is connected;

**3** **for** *spanning trees* S∈S **do**

**4** density ← 0;

**5** **for** 1 ≤ *k* ≤ *i* **do**

**6**  **for** *u*, *v* ∈ *V*(*G*) *with* *d*(*u*, *v*) = *k* **do**

**7**   $\text{density}\;\leftarrow \;density+({\left(deg\left(u\right)\right)}^{j_{k}}+{\left(deg\left(v\right)\right)}^{j_{k}})$;

**8**  **end**

**9** **end**

**10** *D*_*S*_ ← density;

**11** **end**

**12** Find S∈S with the maximum *D*_*S*_;

**13** *w* ← 0;

**14** **for** *u*, *v* ∈ *V*(*S*) **do**

**15** *w* ← *w* + *d*(*u*, *v*);

**16** **end**

**17** Return *S* and *w*;

Before ending this section we want to point out that the above algorithm can be easily adapted to analyze similar problems, motivated from variations of MST (mentioned in the introduction):

- the *k*-DST (finding the densest subtree that contains exactly *k* vertices);
- the Steiner DST: Given a set of terminals, find the densest subtree that connects them;
- degree constrained DST;
- capacitated (see [4] for details) DST;
- DST with conflict pairs (i.e. pairs of vertices are given such that at most one of each pair can be chosen in the spanning tree);
- the problem of finding dense (spanning) subgraph such as unicyclic graphs and bicyclic graphs.

## Concluding remarks

By employing the sum of vertex degrees at different distances and assigning different exponents to the degrees before summation to solve the DST, we are able to analyze the roles of different degree sums play in identifying dense subtrees. While the adjacent degree sum, equivalent to the sum of squares of degrees, is obviously not the optimal condition alone, it is indeed an important part of measuring the denseness of a spanning tree or subgraph in general. Our analysis shows that when

$$\begin{array}{c} \vec{j}=\left(4,2,0,0\right)\;\text{or}\;\left(4,2,2,0\right)\;\text{or}\;\left(4,2,2,2\right) \end{array}$$

the corresponding conditions

$$\begin{array}{c} \sum_{d(u,v)=1}({\left(deg\left(u\right)\right)}^{4}+{\left(deg\left(v\right)\right)}^{4})+\sum_{d(u,v)=2}({\left(deg\left(u\right)\right)}^{2}+{\left(deg\left(v\right)\right)}^{2})+2\cdot \ell_{3}+2\cdot \ell_{4}, \end{array}$$

$$\begin{array}{l} \sum_{d\left(u,v\right)=1}\left({\left(deg\left(u\right)\right)}^{4}+{\left(deg\left(v\right)\right)}^{4}\right)+\sum_{d\left(u,v\right)=2}\left({\left(deg\left(u\right)\right)}^{2}+{\left(deg\left(v\right)\right)}^{2}\right)\\ \;+\sum_{d\left(u,v\right)=3}\left({\left(deg\left(u\right)\right)}^{2}+{\left(deg\left(v\right)\right)}^{2}\right)+2\cdot l_{4} \end{array}$$

and

$$\begin{array}{l} \sum_{d\left(u,v\right)=1}\left({\left(deg\left(u\right)\right)}^{4}+{\left(deg\left(v\right)\right)}^{4}\right)+\sum_{d\left(u,v\right)=2}\left({\left(deg\left(u\right)\right)}^{2}+{\left(deg\left(v\right)\right)}^{2}\right)\\ \;+\sum_{d\left(u,v\right)=3}\left({\left(deg\left(u\right)\right)}^{2}+{\left(deg\left(v\right)\right)}^{2}\right)+\sum_{d\left(u,v\right)=4}\left({\left(deg\left(u\right)\right)}^{2}+{\left(deg\left(v\right)\right)}^{2}\right) \end{array}$$

appear to be the most effective when solving DST for graphs with no more than 50 vertices. Here *ℓ*_*i*_ is the number of pairs of vertices at distance *i* from each other.

As future work, we intend to make use of the identification of these optimal degree sum conditions to find faster and more precise heuristics for DST. Furthermore, while we measured the denseness of a spanning tree through the Wiener index and number of subtrees (as discussed in the introduction), it will be interesting to see how other related graph invariants behave in our dense spanning trees. One of the most interesting such concepts is the Wiener polarity index, defined as the number of pairs of vertices at distance 3 from each other. Also introduced by Wiener, this concept contains information from both distance and substructures (as in a spanning tree each pair of vertices induces a unique path). Consequently, another potential topic for future research is to study the behavior of the Wiener polarity index in the dense spanning trees and compare the results with the most recent work such as [18] and [19].

## Supporting information

S1 File Supporting data. (PDF)

## References

[1] Bazlamaçci C, Hindi K. Minimum-weight spanning tree algorithms:A survey and empirical study, Computers and Operations Research. 2001 28, 767–785. 10.1016/S0305-0548(00)00007-1

[2] Hwang FK, Richards DS, Winter P. The steiner tree problem. North-Holland, New York (1992).

[3] Narula SC, Ho CA. Degree-constrained minimum spanning tree. Comput. Oper. Res. 1980 Vol. 7, pp. 239–249. 10.1016/0305-0548(80)90022-2

[4] Amberg A, Domschke W, Voß S. Capacitated minimum spanning trees: algorithms using intelligent search. Combinatorial Optimization: Theory and Practice. 1996 Vol. 1 9–40.

[5] Darmann A, Pferschy U, Schauer J. Minimal spanning trees with conflict graphs. Optimization online, 2009.

[6] Wiener H. Structural determination of paraffin boiling points. J. Am. Chem. Soc. 1947 69, 17–20. 10.1021/ja01193a005 20291038

[7] Wiener H. Correlation of heats of isomerization, and differences in heats of vaporization of isomers among the paraffin hydrocarbons. J. Am. Chem. Soc. 1947 69, 2636–2638. 10.1021/ja01203a022

[8] Dobrynin AA, Entringer R, Gutman I. Wiener index of trees: Theory and applications. Acta Appl. Math. 2001 66, 211–249. 10.1023/A:1010767517079

[9] Székely LA, Wagner S, Wang H. Problems related to graph indices in trees Recent trends in combinatorics, 3–30, IMA Vol. Math. Appl., 159, Springer, [Cham], 2016.

[10] Székely LA, Wang H. On subtrees of trees. Adv. Appl. Math. 2005 34, 138–155. 10.1016/j.aam.2004.07.002

[11] Wagner S. Correlation of graph-theoretical indices. SIAM J. Discrete Mathematics. 2007 21(1), 33–46. 10.1137/050631446

[12] Knudsen B. Optimal multiple parsimony alignment with affine gap cost using a phylogenetic tree Lecture Notes in Bioinformatics. 2003 2812, Springer Verlag, 433–446.

[13] Jamison RE. On the average number of nodes in a subtree of a tree. J. Combin. Theory Ser. B. 1983 35(3), 207–223. 10.1016/0095-8956(83)90049-7

[14] Jamison RE. Monotonicity of the mean order of subtrees. J. Combin. Theory Ser. B. 1984 37(1), 70–78. 10.1016/0095-8956(84)90046-7

[15] Ozen M, Wang H, Wang K, Yalman D. An edge-swap heuristic for finding dense spanning trees. Theory Appl. Graphs. 2016 3 no. 1, Art. 1, 10 pp.

[16] Ozen M, Wang H, Yalman D. Finding dense spanning trees through integer linear programming. preprint.

[17] Chandler C. Network Modeling of Infectious Disease: Transmission, Control and Prevention (2017) Georgia Southern University Honors Program Theses. 258.

[18] Ma J, Shi Y, Wang Z, Yue J. On Wiener polarity index of bicyclic networks. Sci. Rep. 2016 6, 19066 10.1038/srep19066 26750820 PMC4707490

[19] Lei H, Li T, Shi Y, Wang H. Wiener polarity index and its generalization in trees. MATCH Commun. Math. Comput. Chem. 2017 78(1), 199–212.

