# Supplementary material for: Degree sums and dense spanning trees
Source: PLoS One. 2017 Sep 19;12(9):e0184912. doi: 10.1371/journal.pone.0184912 (PMC5605090; doi:10.1371/journal.pone.0184912)
Supplement: S1 File — (PDF) [file pone.0184912.s001.pdf]

Fig 1. Comparison between $\vec{j}$ = (1, 0, 0), (0, 1, 0), (0, 0, 1).

300 random graphs with 6 vertices

	(1,0,0)	(0,1,0)	(0,0,1)
25	170	0	0
28	130	0	0
29	0	253	0
31	0	47	0
32	0	0	300

600 random graphs with 7 vertices

	(1,0,0)	(0,1,0)	(0,0,1)
36	348	0	0
40	229	1	0
42	21	542	0
44	0	21	0
46	2	36	24
48	0	0	576

300 random graphs with 8 vertices

	(1,0,0)	(0,1,0)	(0,0,1)
49	187	0	0
54	113	0	0

58	0	278	0
62	0	22	0
66	0	0	300

Fig 2. Comparison between $\vec{j}$ = (2; 0; 0); (0; 2; 0); (0; 0; 2) (top) and $\vec{j}$= (0:5; 0; 0); (0; 0:5; 0); (0; 0; 0:5) (bottom).

300 random graphs with 6 vertices, $\vec{j}$ = (2; 0; 0); (0; 2; 0); (0; 0; 2)

	(2,0,0)	(0,2,0)	(0,0,,2)
25	170	0	0
28	130	0	0
29	0	300	0
32	0	0	300

600 random graphs with 7 vertices, $\vec{j}$ = (2; 0; 0); (0; 2; 0); (0; 0; 2)

	(2,0,0)	(0,2,0)	(0,0,2)
36	348	0	0
40	229	1	0
42	21	597	24
44	0	2	0
46	2	0	0
48	0	0	575
50	0	0	1

300 random graphs with 8 vertices, $\vec{j}$ = (2; 0; 0); (0; 2; 0); (0; 0; 2)

	(2,0,0)	(0,2,0)	(0,0,2)
49	187	0	0
54	113	0	0
57	0	6	0
58	0	294	0
66	0	0	300

300 random graphs with 6 vertices, $\vec{j}$= (0:5; 0; 0); (0; 0:5; 0); (0; 0; 0:5)

	(0.5,0,0)	(0,0.5,0)	(0,0,0.5)
25	170	0	0
28	130	0	0
31	0	300	0
32	0	0	300

600 random graphs with 7 vertices, $\vec{j}$= (0:5; 0; 0); (0; 0:5; 0); (0; 0; 0:5)

	(0.5,0,0)	(0,0.5,0)	(0,0,0.5)
36	348	0	0
40	229	0	0
42	21	10	0
46	2	188	8

48	0	402	592

300 random graphs with 8 vertices, $\vec{j}$= (0:5; 0; 0); (0; 0:5; 0); (0; 0; 0:5)

	(0.5,0,0)	(0,0.5,0)	(0,0,0.5)
49	187	0	0
54	113	0	0
62	0	300	0
66	0	0	300

Fig 3. Comparison between $\vec{j}$= (2; 2; 0; 0), (2; 2; 1; 0), and (2; 0; 0; 0).

331506 random graphs with 7 vertices

	(2,2,0,0)	(2,2,1,0)	(2,0,0,0)
36	9555	9555	9555
40	110691	110691	110691
42	123060	123060	123060
44	77490	77490	41484
46	10710	10710	44260
48	0	0	2456

Fig 4. Statistics corresponding to conditions with $\vec{j}$ = (1; 1; 1; 1), (1; 1; 1; 2), and (2; 2; 2; 4).

331506 random graphs with 7 vertices

	(1,1,1,1)	(1,1,1,2)	(2,2,2,4)

36	1590	35	9555
40	21134	126	651
42	25366	0	0
44	58159	0	2016
46	149873	70	0
48	75384	0	0
50	0	3570	0
52	0	327705	318780
56	0	0	504

Fig 5. Statistics corresponding to conditions with $\vec{j}$ = (4; 2; 0; 0), (4; 2; 2; 0), (2; 4; 0; 0),and (2; 4; 2; 0).

More than 2000 random graphs with 11, 12, 13, 14, 15, 16, 17 vertices

	(4,2,0,0)	(4,2,2,0)	(2,4,0,0)	(2,4,2,0)
100	52	52	0	0
108	154	154	22	22
114	72	72	256	256
121	52	52	0	0
122	22	22	22	22
130	154	154	22	22
137	72	72	0	0
142	0	0	256	256
144	64	64	0	0

146	22	22	22	22
154	164	164	20	20
162	56	56	0	0
168	0	0	264	264
169	64	64	0	0
172	16	16	16	16
180	164	164	20	20
189	56	56	0	0
196	64	64	264	264
200	16	16	16	16
208	153	153	17	17
218	69	69	0	0
225	64	64	0	0
226	0	0	269	269
230	14	14	14	14
238	153	153	17	17
249	69	69	0	0
256	64	64	0	0
258	0	0	269	269
262	14	14	14	14
270	153	153	17	17
282	69	69	0	0

296	14	14	14	14
300	0	0	269	269
